# Exercise-induced vasospastic angina diagnosed with a hand grip test in the catheterization laboratory: a case report

**DOI:** 10.1093/ehjcr/ytad638

**Published:** 2024-01-05

**Authors:** Yoshiaki Kawase, Takayuki Warisawa, Kento Kikuchi, Takuya Mizukami, Hitoshi Matsuo

**Affiliations:** Department of Cardiology, Gifu Heart Center, 4-14-4 Yabutaminami, Gifu 500-8384, Japan; Department of Cardiology, Gifu Heart Center, 4-14-4 Yabutaminami, Gifu 500-8384, Japan; Department of Cardiology, NTT Medical Center Tokyo, Tokyo, Japan; Department of Cardiology, St. Marianna University School of Medicine, Kawasaki, Japan; Department of Cardiology, Gifu Heart Center, 4-14-4 Yabutaminami, Gifu 500-8384, Japan; Department of Cardiology, Gifu Heart Center, 4-14-4 Yabutaminami, Gifu 500-8384, Japan; Department of Cardiology, Gifu Heart Center, 4-14-4 Yabutaminami, Gifu 500-8384, Japan

**Keywords:** Exercised-induced, Vasospastic angina, Hand grip test, Angiography, Case report

## Abstract

**Background:**

Exercise-induced vasospastic angina (VSA) is a relatively uncommon clinical scenario and is difficult to diagnose in the catheterization laboratory.

**Case summary:**

A 61-year-old Japanese man presented to our hospital with complaints of angina upon exertion in the morning. Neither a 12-lead electrocardiogram nor an echocardiogram showed any abnormal findings. Invasive coronary angiogram revealed moderate stenosis in the left anterior descending coronary artery. A hand grip test was performed, during which the patient experienced chest pain, and coronary angiogram showed coronary spasm at the site of organic stenosis with delayed coronary flow. Intracoronary nitrates (300 ug) were administered, resulting in the release of coronary spasm.

**Conclusion:**

The hand grip test may serve as a useful method for diagnosing exercise-induced VSA in the catheterization laboratory.

**Learning points**

- A hand grip test, especially in the morning, is useful for the diagnosis of exercise-induced vasospastic angina in the catheterization laboratory.
- Exercise-induced vasospasm might be one of the mechanisms behind the significant diurnal variation in exercise tolerance of patients with vasospastic angina.

## Introduction

The diagnosis of exercise-induced vasospastic angina (VSA) can be challenging, as the classic presentation of VSA is rest angina.^1^ However, there are cases that cannot be explained without considering the concept of exercise-induced VSA in daily practice.^2,3^ One important characteristic of VSA is the marked diurnal variation in exercise tolerance.^1^ It is unclear whether this variation is solely due to the presence of severe coronary artery spasm, which can cause ischaemia with exercise or if exercise itself triggers coronary artery spasm in the morning.

## Summary figure

**Figure:**
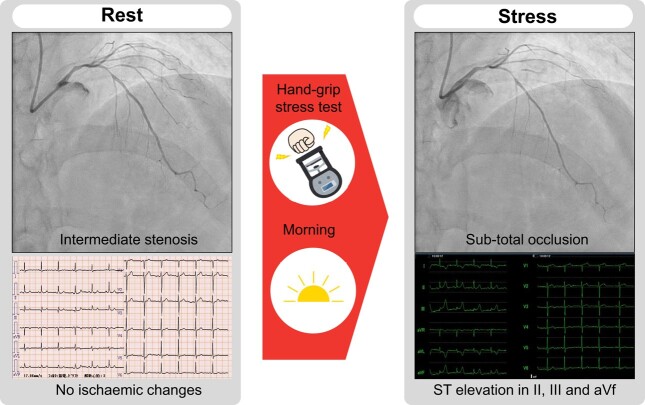


## Case presentation

**Figure 1:**
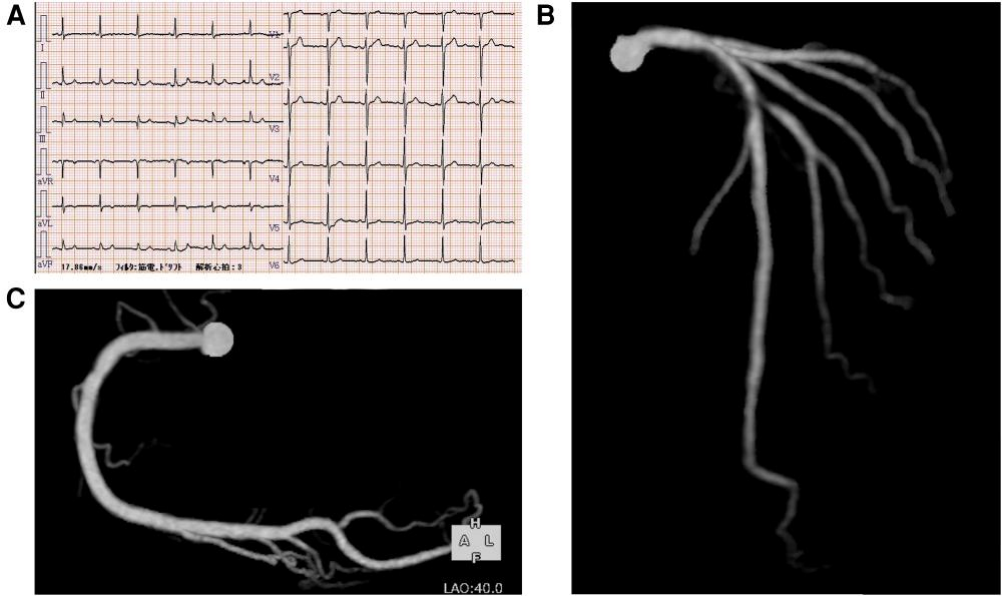
(*A*) A 12-lead electrocardiogram showing no abnormal findings. (*B*) Computed tomography coronary angiogram of the left anterior descending coronary artery, revealing moderate organic stenosis in the proximal part. (*C*) Computed tomography coronary angiogram of the right coronary artery, indicating the absence of organic stenosis.

**Figure 2:**
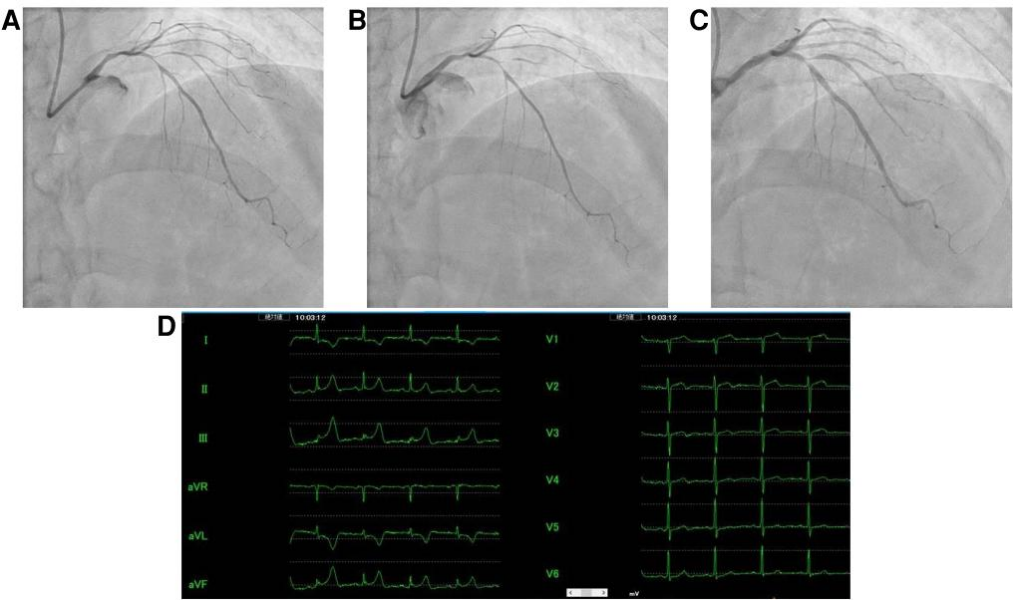
(*A*) Baseline invasive coronary angiogram, illustrating a spastic left coronary with organic stenosis at the proximal part of the left anterior descending coronary artery and diagonal branch. (*B*) Invasive angiogram after a hand grip test, showing an increased severity of stenosis in the proximal part of the left anterior descending coronary artery and diagonal branch. The flow distal to the diagonal branch is delayed. (*C*) Invasive angiogram after the administration of nitrate, indicating a dilated coronary artery. There is a residual organic stenosis in the proximal part of left anterior descending coronary artery and diagonal branch. (*D*) A 12-lead electrocardiogram taken at the end of the hand grip test, revealing elevated ST segments in leads II, III, and aVF.

A 61-year-old Japanese man with multiple coronary risk factors, including diabetes mellitus, hypertension, dyslipidaemia, a family history of coronary artery disease, and current smoking, presented to our hospital with a history of angina upon exertion. Imeglimin hydrochloride 2000 mg, candesartan cilexetil 8 mg, empagliflozin 25 mg, linagliptin 5 mg, and pitavastatin calcium 2 mg were prescribed to him by his primary care physician. The patient experienced episodes of chest pain (Canadian Cardiovascular Society Angina Grade II) predominantly in the morning, which typically resolved within 3–5 min after cessation of exercise. His blood pressure was 116/70 mmHg and heart rate was 88 b.p.m. No cardiac murmur was audible, and no leg oedema was observed on physical examination. The serum N-terminal pro-brain natriuretic peptide level was 13 pg/mL (normal range, 0–125 pg/mL), the low-density lipoprotein cholesterol level was 151 mg/dL (normal range, 70–139 mg/dL), and glycated haemoglobin (HbA1c) was 6.8% (normal range, <6.0%). The chest X-ray showed neither cardiomegaly nor pulmonary congestion. Both the electrocardiogram (*Figure 1A*) and transthoracic echocardiogram showed no abnormal findings. A computed tomography coronary angiogram revealed a moderate stenosis at the proximal site of the left anterior descending coronary artery and diagonal coronary artery (*Figure 1B*) with no stenosis in the right coronary artery (*Figure 1C*). Invasive coronary angiogram was scheduled. Given the clustering of chest pain in the morning, VSA with fixed stenosis was suspected. The baseline coronary angiogram revealed a relatively spastic coronary artery, which describes the condition of coronary artery with relatively even and diffuse reductions in vessel diameter along the coronary artery in angiography (*Figure 2A*). Since the patient complained of angina upon exertion, we initially performed a hand grip test (20 kg × 5 min: 50% of his maximum grip strength). At the end of the 5 min hand grip test, an increase in heart rate and blood pressure was observed, and the patient reported chest pain accompanied by prominent ST elevation in leads II, III, and aVF (*Figure 2D*). Angiogram revealed spasm at the site of the organic stenosis with delayed flow in his diagonal coronary artery (*Figure 2B*). Nitrate (300 ug) was administered to the left coronary artery as the patient’s symptoms were uncontrollable. Subsequent coronary angiography of the right coronary artery, performed immediately after nitrate administration, showed already dilated right coronary artery. Following nitrate administration, the left coronary artery angiogram showed residual organic stenosis in the proximal part of the left anterior descending coronary artery and the diagonal branch (*Figure 2C*). Physiological assessment of the moderate lesion in the proximal part of the left anterior descending coronary artery and the diagonal branch was performed using a PressureWire™ X (Abbott, USA) and intracoronary administration of 2 mg nicorandil. Fractional flow reserve of 0.86 (normal range, >0.80) in the far distal part of the left anterior descending coronary artery and 0.84 in the far distal part of the diagonal branch confirmed the absence of ischaemia caused by the organic stenosis. With reproducible angina during the provocation test, ST segment changes in electrocardiogram, and spasm at the site of organic stenosis, exercise-induced VSA was diagnosed. A calcium channel blocker was administered, and smoking cessation guidance was provided at discharge. Nitrate was used as needed, since the patient was intolerant for the regular use of long-acting nitrate due to headache. Since then, the patient has been free from angina.

## Discussion

The relationship between typical VSA patients, who experience chest symptoms at rest, and exercise-induced ST elevation has been extensively documented.^4–7^ While the ergonovine (ergometrine), a medication originally used to induce contraction of smooth muscle tissue in the blood vessels for the treatment of heavy vaginal bleeding after childbirth, test has demonstrated higher sensitivity in diagnosing VSA,^7^ there is a paucity of studies comparing these diagnostic methods in patients predominantly experiencing exertional angina^4,8^ (*Table 1*). This gap gives rise to uncertainties regarding the suitability of the acetylcholine provocation test for accurately diagnosing such cases. Notably, within Waters’s^7^ study, a case was observed where ST elevation occurred during the exercise test but not during the acetylcholine provocation test in a typical VSA patient. However, performing exercise testing in patients with VSA poses a potential risk of lethal arrhythmias. High rates of ventricular fibrillation incidence (1.8–17%) have been reported with exercise-induced testing in VSA patients^4,9^ (*Table 1*). Conducting the exercise test within the catheterization laboratory setting could offer preparatory measures to counter potential haemodynamic instability, as recently reported by Tamura *et al.*^2^ The difficulty of performing an exercise stress test in the catheterization laboratory poses a problem in diagnosing exercise-induced VSA.^8,9^ A hand grip test can serve as a relatively easy and cost-effective alternative to a supine cycle ergometer.^10^ However, concerns arise regarding whether the hand grip test can provide sufficient stress compared with the cycle ergometer. Peak levels of oxygen consumption, heart rate, and cardiac output are higher during maximal isotonic exercise compared with maximal isometric exercise, and the combination with infusion of dipyridamole is suggested for detecting the presence of organic stenosis when using isometric exercise.^6^ However, it is important to note that provoking spasm at the lesion site may differ from detecting ischaemia caused by a stenotic lesion. Furthermore, as mentioned earlier, patients with VSA often exhibit a marked diurnal variation in exercise tolerance. Performing the hand grip test in the morning may already be a combined method for inducing coronary artery spasm in patient with VSA.^5^ In fact, in the present case, we performed the hand grip test early in the morning in the catheterization laboratory rooms, which clearly and safely demonstrated the utility of the hand grip test in provoking coronary spasm.

**Table 1:** Summary of evidence from exercise testing in patients with vasospastic angina

Authors	Year	Journal	Type of chest pain	Evidence
Detry *et al.*	1975	Br Heart J	At rest (*n* = 2), at rest and exertion (*n* = 4)	The prevalence of life-threatening arrhythmia in patient with vasospastic angina who displayed ST elevation during maximal exercise testing. The reported incidence of ventricular fibrillation was 17%
Waters *et al.*	1979	Circulation	At rest (*n* = 7)	The reproducibility of exercise-induced ST elevation in patient with vasospastic angina
Yasue *et al.*	1979	Circulation	Early morning or night (*n* = 13)	Circadian variation of exercise-induced ST elevation in patient with vasospastic angina
de Servi *et al.*	1981	Circulation	At rest (*n* = 114)	The prevalence of coronary artery disease in patients with vasospastic angina who exhibited varying responses to exercise testing. The incident of ventricular fibrillation was reported to be 1.8%
Specchia *et al.*	1981	Circulation	At rest (*n* = 5), at exertion (*n* = 2), both (*n* = 9)	The type of chest pain observed in daily life among patients who display ST elevation during exercise testing
Waters *et al.*	1982	Circulation	At rest (*n* = 82)	The correlation between disease activity and sensitivity of exercise testing for diagnosing patients with vasospastic angina
Waters *et al.*	1983	Circulation	At rest (*n* = 34)	The sensitivity of ergonovine, cold pressor, and exercise testing for diagnosing patients with vasospastic angina

## Supplementary Material

ytad638_Supplementary_Data

## Data Availability

The data underlying this article will be shared on reasonable request to the corresponding author.

## Lead author biography

Yoshiaki Kawase is an interventional cardiologist who graduated from Gifu University School of Medicine, Gifu, Japan, in 1998. Since 2010, he has been serving as a co-director of the catheterization laboratory at Gifu Heart Center in Gifu, Japan.
